# Internet-based cognitive bias modification for obsessive compulsive disorder: study protocol for a randomized controlled trial

**DOI:** 10.1186/1745-6215-15-193

**Published:** 2014-05-29

**Authors:** Alishia D Williams, Rosanna Pajak, Kathleen O’Moore, Gavin Andrews, Jessica R Grisham

**Affiliations:** 1 School of Psychiatry, UNSW Medicine, University of New South Wales, Sydney, NSW 2052, Australia; 2 Clinical Research Unit for Anxiety and Depression (CRUfAD), St. Vincent’s Hospital, 394-404 Victoria Street, Darlinghurst, NSW 2010, Australia; 3 School of Psychology, UNSW Faculty of Science, University of New South Wales, Sydney, NSW 2052, Australia

**Keywords:** cognitive-bias modification, CBM, obsessive compulsive disorder, OCD, randomized controlled trial, RCT, interpretation bias

## Abstract

**Background:**

Cognitive bias modification (CBM) interventions have demonstrated efficacy in augmenting core biases implicated in psychopathology. The current randomized controlled trial (RCT) will evaluate the efficacy of an internet-delivered positive imagery cognitive bias modification intervention for obsessive compulsive disorder (OCD) when compared to a control condition.

**Methods/Design:**

Patients meeting diagnostic criteria for a current or lifetime diagnosis of OCD will be recruited via the research arm of a not-for-profit clinical and research unit in Australia. The minimum sample size for each group (alpha set at 0.05, power at .80) was identified as 29, but increased to 35 to allow for 20% attrition. We will measure the impact of CBM on interpretations bias using the OC Bias Measure (The Ambiguous Scenarios Test for OCD ;AST-OCD) and OC-beliefs (The Obsessive Beliefs Questionnaire-TRIP; OBQ-TRIP). Secondary outcome measures include the Dimensional Obsessive-Compulsive Scale (DOCS), the Patient Health Questionnaire (PHQ-9), The Kessler Psychological Distress Scale (K10), and the Word Sentence Association Test for OCD (WSAO). Change in diagnostic status will be indexed using the OCD Mini International Neuropsychiatric Interview (M.I.N.I) Module at baseline and follow-up. Intent-to-treat (ITT) marginal and mixed-effect models using restricted maximum likelihood (REML) estimation will be used to evaluate the primary hypotheses. Stability of bias change will be assessed at 1-month follow-up.

**Discussion:**

A limitation of the online nature of the study is the inability to include a behavioral outcome measure.

**Trial registration:**

The trial was registered on 10 October 2013 with the Australian New Zealand Clinical Trials Registry (ACTRN12613001130752)

## Background

Cognitive bias modification is a cognitive experimental methodology that modifies cognitive biases via training conditions, in which participants are exposed to a series of stimuli designed to manipulate processing relevant to psychopathology. CBM interventions are either designed to modify attentional bias (CBM-A) or interpretive bias (CBM-I). Both forms have been shown to impact clinically relevant symptoms across a range of anxiety disorders and depression with effect sizes for CBM-I on biases ranging up to Hedges *g* = .81 (see reviews by Beard [1]; Hallion and Ruscio [2]; Macleod [3] Macleod and Matthews [4]). CBM-I interventions have demonstrated efficacy in modifying the key cognitive biases implicated in various anxious populations, including high trait anxiety [5,6], generalized anxiety [7], spider fear [8] and social anxiety [9-13]. This research has also extended to include transdiagnostic constructs such as perfectionism, which is associated with a range of clinical diagnoses [14]. Importantly, some studies have shown that the resultant change in selective information processing due to CBM-I interventions impacts emotional responses to subsequent stressor tasks [7,14-17], as well as relevant anxiety symptoms [12,13,16].

CBM-I procedures may have particular relevance in the context of obsessive compulsive disorder (OCD), given that prominent cognitive models of the disorder assert that intrusive thoughts are experienced by most people, but develop into obsessions when appraised (interpreted) as posing a threat for which the individual is personally responsible. Research has identified a series of belief domains from which these negative interpretations arise [18] (OCCWG, 2005): overestimation of threat, inflated responsibility, perfectionism, intolerance of uncertainty, importance of thoughts, and the need to control thoughts. Correlational evidence supports the association between biased interpretations of intrusive thoughts and obsessive-compulsive symptoms [18-21]. Prospective studies have even demonstrated that negative interpretation of intrusive thoughts at baseline predicts OCD symptom severity at follow-up [22,23].

Currently, cognitive-behavioral therapy (CBT) is the primary treatment of OCD. However, response rates are only 50% when drop-out rates are considered [24], implying a clear need for treatment advancements. CBM-I procedures are gaining increasing research attention in association with OCD and high OC symptoms, with evidence now emerging for their impact on interpretation biases, emotional and physiological responses to OC stressors, and subsequent behavior (detailed below). In addition, CBM-I interventions for OCD have been shown to effect change specifically by altering contingency learning about potential threat cues [25], rather than via habituation of fear and arousal as hypothesized for exposure-based interventions.

The most common CBI-I method - utilized in the current study - involves ‘ambiguous scenario training’. Developed by Matthews and Mackintosh [26], the method involves presenting participants with a serious of scenarios that are ambiguous in terms of potential threat. Each scenario ends with a word fragment, which the participant is asked to complete in order to resolve the ambiguity in either a positive (non-threatening) or negative (threatening) way. Repeatedly pairing a potential anxiety trigger with a benign interpretation is proposed to train contingencies between ambiguous (potentially threatening) cues and positive interpretations. This particular method has been used to effect change in interpretation bias across various anxious samples [5,8,11,27].

Clerkin and Teachman [28] first applied this method to obsessive-compulsive (OC) beliefs, evaluating whether participants high in OC-symptoms could be trained to adopt healthier interpretations of OC-related information, across a variety of OC-relevant domains. They also tested whether the ambiguous scenario training influenced participants’ responses to an OC-stressor task (designed to elicit distress and the urge to engage in a compulsion or neutralization behavior). As hypothesized, participants in the positive CBM-I training condition endorsed healthier OC-relevant interpretations (Cohen *d* = .76) and beliefs (*η*^2^*p* = .07) following training versus those in a neutral CBM-I control condition (in which half the scenarios resolved positively and half resolved consistently with OC-beliefs). Those in the positive training condition also reported less negative emotion (at trend level) during the subsequent stressor task, providing initial evidence that modifying interpretation biases in a high OC-population may have downstream effects on emotional and physiological responses to OC-stressors.

To extend these results, Grisham *et al*. [29] used CBM-I to specifically target and modify interpretive bias reflecting inflated responsibility in a sample of undergraduate students with high levels of checking symptoms. Participants were randomly assigned to a Positive (decreasing responsibility interpretations) or Negative (increasing responsibility interpretations) training condition. Although there were no differential effects with respect to observed or self-reported checking or self-reported responsibility beliefs, participants in the Positive training condition did demonstrate reduced responsibility bias in a subsequent interpretive bias test (ηp^2^ = .07), as well as more adaptive physiological responding during a responsibility stressor task (ηp^2^ = .12), compared to the Negative training condition. In a broader study, Williams and Grisham [30] evaluated a CBM-I training paradigm in a sample of community members with varying levels of OC symptoms. The impact of CBM was assessed using measures of interpretation bias, distress, and responses to three behavioral tasks designed to tap the core belief domains implicated in OCD: importance of thoughts/control, perfectionism/intolerance of uncertainty, and contamination/estimation of threat. Participants randomly assigned to the active CBM condition evidenced a change in interpretation bias, endorsing more positive and less negative OC-relevant interpretations compared to participants assigned to the CBM control condition (Cohen’s *d* ES = .66). Importantly, there was not a corresponding shift in interpretive bias to foil scenarios (those unrelated to the core OC belief domains), suggesting specificity in the CBM-I training effect rather than a general positivity bias. Similar to earlier findings [29], there was no significant difference in terms of behavioral responses to the OC stressor tasks, but participants in the Positive condition did report significantly less distress and urge to neutralize following the OC stressor task designed to tap importance of thoughts/control.

Based on these collective results, CBM procedure may hold promise as a means to selectively target OC beliefs, reduce interpretive biases, and lead to changes in emotional responses associated with OC symptoms. However, these results need to be replicated in clinical samples, with adequate follow-up, in order for potential therapeutic benefits to be demonstrated for OCD.

The current SPIRIT-compliant [31] protocol (see Table 1) outlines the methodology of a randomized controlled trial (RCT) to establish the efficacy of a CBM intervention for participants who meet current diagnostic status for OCD when compared to an active CBM control condition. In order to investigate whether previously symptomatic individuals demonstrate a negative interpretation bias for OC-specific information, participants with a lifetime diagnosis of OCD will also be recruited for secondary analyses.

**Table 1:** Items from the World Health Organization trial registration data set

**Secondary identifying numbers**	**ACTRN12613001130752**
Source(s) of monetary or material support	
Primary sponsor	St Vincent’s Hospital, Sydney
Secondary sponsor(s)	
Contact for public queries	AW, GA
Contact for scientific queries	AW, GA
Public title	Cognitive Bias Modification (CBM) and Obsessive Compulsive Beliefs
Scientific title	Randomized controlled trial comparing Internet-based cognitive bias modification (active version) for obsessive compulsive disorder versus Internet based cognitive bias modification (control version) on negative interpretation bias.
Countries of recruitment	Australia
Health condition(s) or problem(s) studied	Obsessive Compulsive Disorder
Intervention(s)	Experimental: CBM Version A (Active treatment)
	CBM Version A is an Internet-based intervention taking place over 1 week.
	Placebo Comparator: CBM Version B (Control)
	CBM Version B (Control) is an Internet-based intervention taking place over 1 week (identical to CBM Version A without the putative active components).
Key inclusion and exclusion criteria	Inclusion: 18 to 65 years of age; Meet Diagnostic and Statistical Manual of the American Psychiatric Association - 4th edition (DSM-IV) criteria for current or lifetime Obsessive Compulsive Disorder; Internet and printer access; Australian resident; fluent in written and spoken English
	Exclusion: Current substance abuse/dependence; Psychotic mental illness (Bipolar or Schizophrenia); change in medication or psychological treatment during last 1 month or intended change during study duration; use of Benzodiazepines, severe depression (PHQ9 > 23), Suicidal (PHQ9 item 9 > 2)
Study type	Interventional
Date of first enrollment	24 October 2013
Target sample size	70
Recruitment status	Recruiting
Primary outcome(s)	Change in: Interpretations bias (the Ambiguous Scenarios Test for OCD; AST-OCD), the Word Sentence Association Test for OCD (WSAO), and the OC Bias measure.
Key secondary outcomes	Change in: The Obsessive Beliefs Questionnaire-TRIP (OBQ-TRIP), the Patient Health Questionnaire (PHQ-9); the Dimensional Obsessive-Compulsive Scale (DOCS). Change in diagnostic status will be indexed using the OCD M.I.N.I Module at baseline and follow up.

### Hypotheses

For participants who meet diagnostic criteria for current OCD, we predict that CBM active training, relative to CBM control training, will result in a bias towards more positive OC-specific targets and a reduction in OC symptom measures. We further predict that benefits will be maintained at 1-month follow-up. For participants who meet criteria for a lifetime diagnosis of OCD (and do not meet criteria for current OCD) and who evidence a negative interpretation bias at baseline, we predict that CBM active training, relative to CBM control training, will result in a bias towards more positive OC-specific targets (OCD-Bias measure) and bias scores (AST-OCD; WSAO). We do not predict a significant change in measures of OC symptoms (DOCS) in individuals with a lifetime diagnosis.

## Methods/Design

### Trial design

The trial is a randomized controlled superiority trial with two parallel arms.

### Study setting

The Clinical Research Unit for Anxiety and Depression (CRUfAD) is a not-for-profit joint initiative of St. Vincent’s Hospital and the University of New South Wales, School of Psychiatry in Sydney, Australia. CRUfAD specializes in the development, evaluation, and dissemination of evidence-based CBT programs via the Internet. The mode of Internet recruitment and delivery enables potential participants from all Australian states to be eligible to apply for enrollment in the current trial.

### Participants and recruitment

Power calculations were informed by calculation of effect size data from Williams and Grisham [30] and Clerkin and Teachman [28] providing between-group effect sizes of .66 and .76, respectively. The minimum sample size for each group meeting current diagnostic status (alpha set at 0.05, power at .80) was identified as 29, but is set at 35 to hedge against expected attrition of up to 20%. Participants will be recruited via the research arm of a not-for-profit clinical and research unit affiliated with St. Vincent’s Hospital and the University of New South Wales, Australia.

**Figure 1:**
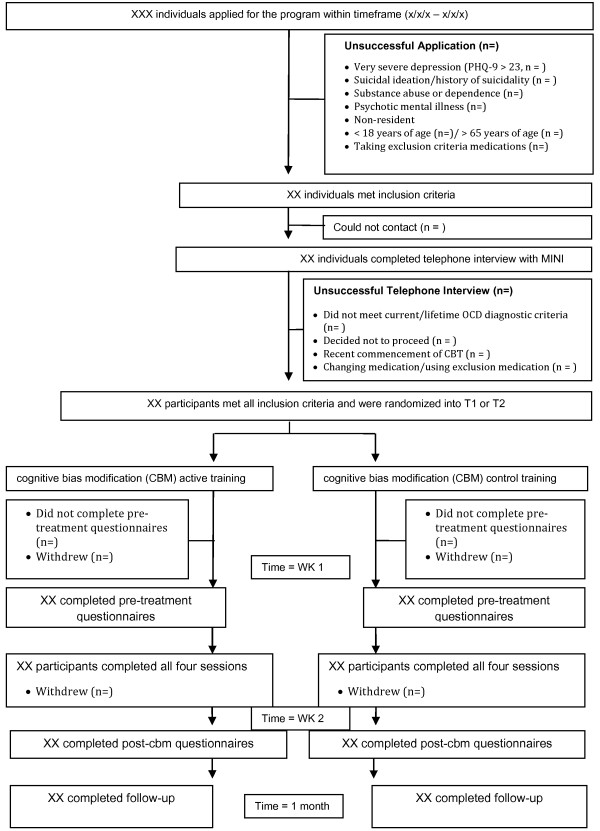
Study flow chart.

Potential participants will be recruited from the community and from previous research trials via advertisements placed on local mental health websites, in newsletters, and in printed newspapers. Applicants will be directed to the Virtual Clinic website (http://www.virtualclinic.org.au) where they first complete automated online screening questionnaires about symptoms and demographic details (see Figure 1). Inclusion criteria are as follows: meet diagnostic criteria for current or lifetime Obsessive Compulsive Disorder; has Internet and printer access; is an Australian resident; and is fluent in written and spoken English. Exclusion criteria are as follows: is a non-resident of Australia; is less than 18 or older than 65 years of age; is currently receiving CBT for OCD; has severe depression and (PHQ9 > 23)/ or suicidal ideation (PHQ9 item 9 = 3); has drug or alcohol dependence; has a psychotic disorder or is taking atypical antipsychotics or benzodiazepines; and if taking medication for anxiety or depression, has been taking the same dose for less than 1 month or intends to change the dose during the course of the program. Excluded applicants receive information on alternative services and are encouraged to discuss their symptoms with their physician.

Applicants whose responses to the questions meet selection criteria are telephoned for a diagnostic interview using the M.I.N.I Version 5.0.0 [32] to determine whether they meet criteria for a current or lifetime diagnosis of Obsessive Compulsive Disorder. Applicants who satisfy all inclusion criteria will be informed of the study design and complete an electronic informed consent prior to being enrolled in the trial. All participants are informed in writing that they have right to withdraw from the study at any time without jeopardizing their relationship with St. Vincent’s Hospital or the University of New South Wales.

### Randomization

Eligible participants accepted into the program will be randomly allocated to either CBM Version A (Group 1 - active condition) or CBM Version B (Group 2 - control condition) based on an allocation sequence generated by an independent person not involved in the study, via a true randomization process (http://www.random.org). Numbers corresponding to treatment group (1 or 2) will be placed in sealed opaque envelopes with the sequential order number written on each envelope to ensure participants are allocated according to the pre-determined sequence. Participants remain blind to treatment group allocation. A member of the research team will open the envelope after all screening procedures have occurred and after the diagnostic interview has been conducted.

### Interventions

#### Cognitive bias modification program (both active and control conditions)

There are four separate sessions of the CBM program, each of approximately 20 to 30 minutes duration. These are completed over the course of five consecutive days (allowing some flexibility to delivery). Both conditions include four days of 164 training scenarios; with each CBM session delivering 41 training scenarios. The CBM training modules were created based on the methodology of Clerkin and Teachman [28] and have been validated in Williams and Grisham [30]. Scenarios are based on the individual items of the OBQ-44 and tap the broad belief domains of Tolerance for Uncertainty, Threat Estimation, Control of Thoughts, Importance of Thoughts, Responsibility and Perfectionism. Participants are asked to read and imagine themselves in various scenarios that could potentiate a negative OC interpretation. Each training scenario is immediately followed by a comprehension question to ascertain compliance with the training instructions. Adherence to the daily CBM sessions will be automatically tracked by the computer program. Standardized email reminders will be sent to a participant if more than two days lapse between logins.

#### Active cognitive bias modification

In this version (the intervention condition) all scenarios have a positive resolution, designed to induce a positive interpretation bias to ambiguous information. For example: ‘You are riding the bus home from work. The passenger beside you sneezes so you offer them a tissue. You think to yourself that offering a tissue was a behavior that was k_nd/’ (requiring the participant to enter the letter ‘i’ to form the word ‘kind’). The scenario completion task was followed by a comprehension question to ensure the participant had processed the meaning of the sentence ‘Are you pleased that you offered a stranger a tissue?’ (YES). In this condition a specific learning contingency is established between the ambiguous start of the scenario and the imagined positive resolution.

#### Control cognitive bias modification

In this version (the control condition) the scenarios are identical in content but are not designed to induce a positive interpretation bias: 50% of the scenarios resolve in a positive manner, 50% of the scenarios resolve in a negative manner. Therefore, no contingency is established between the start of the scenario and the valence of the imagined final resolution. For example, the negative resolution of the scenario above would be as follows: ‘You think to yourself that offering a tissue was a behavior that was r_sky’ (requiring the participant to enter the letter ‘i’ to form the word ‘risky’). The comprehension question corresponding to a negative resolution would be as follows: ‘Are you pleased that you offered a stranger a tissue?’ (NO).

### Procedure

After completion of the informed consent process and enrollment, participants will be sent a link via email to complete the online self-report questionnaires. Participants will then be sent an email with instructions to access the online CBM training modules. Following completion of the final module participants will complete the post-CBM questionnaires (at Day 8). Participants will be contacted after one month to complete the follow-up measures (the primary baseline measures) and telephoned to complete the OCD M.I.N.I Module diagnostic interview.

### Primary outcome measures

#### The Obsessive Compulsive bias measure [28]

To obtain an index of interpretation bias both before and after CBM-I training, the bias measure designed by Clerkin and Teachman [28] will be employed. Participants are first exposed to ten scenarios with a missing letter in the final word of the sentence. Each scenario remains ambiguous in nature even after completion of the word fragment (for example, ‘You are driving to visit friends who live several hours away. Outside, it begins to rain and you are careful to drive the speed limit. You think about the importance of driving s_fely’). In this example, the letter ‘a’ would be required to complete the word stem of ‘safely’. Following the filler task (below), participants are randomly presented with four sentences and required to rate how similar each was to the meaning of the scenario they previously imagined themselves in (1 = very different in meaning to 4 very similar in meaning). Each sentence corresponds with four different interpretations, none of which was worded identically to the sentence in the paragraph they had previously imagined themselves in. OC-positive scenarios are consistent with a response that challenges the core maladaptive belief (for example, ‘As you drive down the road, you think your chances of getting into an accident are low because you are being so cautious’) whereas OC-negative scenarios are those consistent with a response that reinforces the core maladaptive belief (for example, ‘*As you drive down the road, you worry that you’ll accidentally crash your car even though you aren’t speeding’*). Foil scenarios are included to assess for a general interpretation bias. Foil Positive scenarios are positive, but unrelated to core OC maladaptive beliefs (for example, ‘*As you drive down the road, you are looking forward to visiting your friend’*) and Foil Negative scenarios are negative, but also unrelated to OC beliefs (for example, ‘As you drive down the road, you are not looking forward to visiting your friend*’*). Cronbach’s alpha for the indices ranges from .68 to .79. The OC Bias Measure will be administered at baseline, following the 1-week CBM intervention, and at 1-month follow-up.

#### Ambiguous Scenarios Test-obsessive compulsive disorder

The AST-OCD (AW and JG, unpublished work) is a measure of interpretation bias comprising 12 ambiguous scenarios designed to tap the following belief domains: intolerance of uncertainty, perfectionism, threat estimation, control of thoughts, responsibility, and importance of thoughts. Each belief domain comprises two items. Items are rated in terms of their emotional valence (1 = very negative to 7 = very positive) and their associated arousal (1 = very anxious to 7 = very excited). Scores are averaged to form a total score of valence and arousal. Participants are asked to imagine each of the scenarios and imagine the event happening to them (for example, ‘Your partner has taken you to a new restaurant for dinner. Before you eat, you visit the restroom and notice it is unusual’). The AST-OCD will be collected at baseline, following the 1-week CBM intervention, and at 1-month follow-up.

#### The obsessive beliefs questionnaire-TRIP [33]

The OBQ-TRIP (20-item version) is a factor-analytically derived brief version of the original Obsessive Compulsive Cognitions Working Group (OCCWG) 44-item version [18]. Each of the 20 items designed to measure cognitions and beliefs central to OCD are rated on a 7-point Likert-type scale (1 = disagree very much to 7 = agree very much). The OBQTRIP- 20 correlates well with the full OBQ-TRIP and demonstrates good internal consistency, Cronbach’s alpha = .77 to .82 [18]. The OBQ-TRIP will be collected at baseline, following the 1-week CBM intervention, and at 1-month follow-up.

### Secondary Outcome Measures

#### The dimensional obsessive-compulsive scale [34]

The DOCS is a 20-item self-report measure that assesses the severity of the four most consistently replicated OCD symptom dimensions (a) contamination, (b) responsibility for harm and mistakes, (c) symmetry/ordering, and (d) unacceptable thoughts. Items are rated on a 5-point scale, with scores for each symptom dimension range from 0 (minimum) to 20 (maximum) and total scores ranging from 0 to 80. To accommodate the heterogeneity of OCD symptoms, and the presence of obsessions and rituals within each symptom dimension, each subscale begins with a description of the symptom dimension along with examples of representative obsessions and rituals. The examples clarify the form and function of each dimension's fundamental obsessional fears, compulsive rituals, and avoidance behaviors. Within each symptom dimension, five items (rated 0 to 4) assess the following parameters of severity (over the past month): (a) time occupied by obsessions and rituals, (b) avoidance behavior, (c) associated distress, (d) functional interference, and (e) difficulty disregarding the obsessions and refraining from the compulsions. The DOCS demonstrates excellent psychometric properties and the subscales have excellent reliability in clinical samples (*α* = .94 to .96; in current sample *α* = .93 to .96), and the measure converges well with other measures of OC symptoms [34]. The DOCS will be collected at baseline, following the 1-week CBM intervention, and at 1-month follow-up.

#### Patient health questionnaire [35]

The PHQ-9 is a self-report questionnaire corresponding to the DSM-IV diagnostic criteria for major depressive disorder. Each item is rated in frequency on a 4-point (0 = not at all, 3 = nearly every day) scale. Total scores range from 0 to 27 with higher scores reflecting higher levels of psychopathology. A PHQ-9 score of ≥10 is used as a clinical cut-off for probable DSM-IV diagnosis of MDD [36]. The PHQ-9 demonstrates good psychometric properties and has been used extensively to measure treatment outcomes during internet CBT interventions targeting depression and anxiety [37,38]. The PHQ9 will be administered at baseline, following the 1-week CBM intervention and at 1-month follow-up.

#### The word sentence association test for obsessive compulsive disorder [39]

The WSAO is a measure of interpretation bias in OCD, comprising of 20 distinct ambiguous obsessive-compulsive (OC)-related sentences across multiple domains of OC symptoms. Ten sentences are followed by an OC-related threat word and ten are followed by a benign word. Participants are instructed to indicate how related the sentence and the word are to each other. For example, participants see the sentence ‘Part of the floor you are walking on is brown*’* and then circle how related the sentence is to the word ‘excrement’ (threat word). Participants also see ambiguous sentences paired with benign words, such as ‘There were many appliances running when you left*’* and rate how related the sentence is the word ‘busy’. Higher ratings of relatedness for threat words to the ambiguous sentences compared to ratings of relatedness of benign words to the ambiguous sentences reveals a threat interpretation bias on this measure. Internal consistency is good (a = .81) for the total measure. The WSAO will be collected at baseline, following the 1-week CBM intervention, and at 1-month follow-up.

#### Kessler-10 psychological distress scale [40]

The K10 consists of 10 items ranked on a five point scale designed to measure non-specific psychological distress. For the current study, the time-frame was modified to assess psychological distress in the past week rather than in the past 30 days. The K10 possesses strong psychometric properties [40].

#### Diagnostic status

Change in diagnostic status will be indexed using the M.I.N.I. [32] Obsessive Compulsive Disorder (OCD) Module, administered at baseline and 1-month follow-up. All research personnel conducting diagnostic assessments will be blind to the baseline interview and will have received extensive training in administration of the M.I.N.I by a registered psychologist with PhD level qualifications.

### Data collection and management

Data for the primary and secondary outcome questionnaires are collected via the Virtual Clinic system software and Key Survey software licensed by UNSW. All information collected by the Key Survey and CBM software is coded with either a participant identification number or an email address to facilitate data-to-patient matching. Clinical information, including diagnostic status using the M.I.N.I., is collected by interview via telephone and stored in written format in a secure location at CRUfAD. Any identifiable information that is collected remains confidential, except as required by law. Only members of the site (CRUfAD) research team will have access to participant information and data.

To promote participant retention, participants are reminded that data collection is an important aspect of research and enables the research team to track their progress and to evaluate the program. Participants are offered one entry into a lottery for a gift card valued at $100 AUD in exchange for completion of the 1-month follow-up questionnaires. All data will be extracted from the CBM software servers in the form of either an SPSS output file or an Excel compatible file to be transferred to SPSS by a member of the research team. All data will be stored on a secure Virtual Clinic server.

Participants are informed in writing that the research team will plan to publish the results of the trial in peer-reviewed scientific publications and presentations. Participants are informed that in any publication or presentation, information will be provided in such a way to maintain anonymity. All members of the research team who provide intellectual input to the trial design, execution, or write-up will be acknowledged as an author on any publications. Participants will be sent (via email) a written summary of the results in lay terms following completion of the trial study phase.

### Statistical methods

Significance testing of group differences regarding demographic data and pre-treatment measurements will be conducted using ANOVA and χ2 where the variables consist of nominal data. Intent-to-treat (ITT) mixed models using restricted maximum likelihood (REML) estimation will be used to account for missing data due to participant drop-outs. Significant effects will be followed up with pairwise contrasts comparing pre-treatment to post-treatment scores. Complete-case analyses of the primary hypotheses using data from participants who complete all four sessions of the CBM program will also be conducted. The effect of potential treatment moderators will be evaluated by including baseline variables of interest as a covariate and interaction term in separate mixed models analyses. Analyses will be performed in SPSS. Effect sizes will be calculated between groups (Hedges *g*) and within groups (Cohen’s *d*, adjusting for the repeated measures correlation) using the pooled standard deviation and adjusted for sample size.

### Monitoring

The clinical trials manager of CRUfAD and a member of the research team will oversee data collection and monitoring. An interim analysis will only be conducted to check that the planned number of participants have been retained in the trial following enrollment. Any adverse events will be reported to the head of CRUfAD and to the HREC of St. Vincent’s Hospital, the responsible body for initiating a clinical trial audit.

### Ethics and dissemination

The current trial protocol has been approved by the Human Research Ethics Committee (HREC) of St. Vincent’s Hospital and the University of New South Wales, Sydney. The trial is registered as ACTRN12613001130752. No protocol amendments have been made.

## Discussion

The current randomized controlled trial will provide a test of the utility of a CBM intervention in modifying maladaptive negative interpretation biases associated with OCD. The acceptability of CBM as a credible treatment is still unknown. Although a qualitative study suggests CBM-I (targeting social anxiety) is acceptable to patients, the tasks may need refining to enhance engagement [41]. In the current study, outcome measures will focus on interpretative bias and OC symptomology; a limitation of the online nature of the study is the inability to include a behavioral outcome measure (OC-stressor task). While CBM-I interventions have been linked to subsequent reductions in anxiety [28] and more adaptive physiological responding during OC stressor tasks [29], change in behavioral responses has not yet been demonstrated. Reviews such as those by Beard [1] and Hallion and Ruscio [2] argue the importance of future studies including both behavioral and neural outcome measures to ascertain the true scope of CBM-I interventions.

## Trial status

This article was submitted on 6 January 2014. To date, 28 participants have met eligibility requirements and been randomized to treatment condition. The first round of applications opened on 24 October 2013 and the first participant was enrolled on 25 October 2013. Data collection aims to be complete in June 2015.

## Abbreviations

CBM: cognitive bias modification; OCD: obsessive compulsive disorder; ITT: intent-to-treat; AST-OCD: The Ambiguous Scenarios Test for OCD; OBQ-TRIP: the Obsessive Beliefs Questionnaire; DOCS: Dimensional Obsessive-Compulsive Scale, PHQ-9, the Patient Health Questionnaire, K10, the Kessler Psychological Distress Scale; WSAO: the Word Sentence Association Test for OCD; M.I.N.I: Mini International Neuropsychiatric Interview, CBM-A, attentional bias; CBM-I: interpretive bias.

## Competing interests

The authors declare that they have no competing interests.

## Authors’ contributions

AW conceived of the study, prepared the protocol, and initiated the trial. RP contributed to the study design and writing of the protocol. KO contributed to the study design and coordination of the trial. GA contributed to the study design. JG contributed to the study design. All authors contributed to refinement of the study protocol and read and approved the final manuscript.
